# Polarization-dependent photonic crystal fiber optical filters enabled by asymmetric metasurfaces

**DOI:** 10.1515/nanoph-2022-0001

**Published:** 2022-03-17

**Authors:** Indra Ghimire, Jingyi Yang, Sudip Gurung, Satyendra K. Mishra, Ho Wai Howard Lee

**Affiliations:** Department of Physics and Baylor Research and Innovation Collaborative (BRIC), Baylor University, Waco, USA; Department of Physics & Astronomy, University of California, Irvine, CA 92697, USA; Centre for Optics, Photonics, and Lasers (COPL), Université Laval, Quebec, Canada

**Keywords:** metasurface, nanostructure, optical fiber, photonic crystal fiber

## Abstract

We demonstrate in-fiber polarization-dependent optical filter by nanopatterning an asymmetric metallic metasurface array on the end-facet of polarization-maintaining photonic-crystal fibers. The asymmetric cross-typed nanoslit metasurface arrays are fabricated on the core of the optical fiber using the focused ion beam milling technique. Highly polarization- and wavelength-dependent transmission with transmission efficiency of ∼70% in the telecommunication wavelength was observed by launching two orthogonal linear-polarization states of light into the fiber. Full-wave electromagnetic simulations are in good agreement with the experimental results. These advanced meta-structured optical fibers can potentially be used as novel ultracompact in-fiber filters, splitters, and polarization converters.

Optical fibers have proven to be an efficient platform for light guiding with low optical loss, leading to wide range of emerging optical applications such as long distance optical communication [1], fiber lasers [2], in-fiber imaging, sensing, and laser surgery [3], [4], [5], [6], [7], [8]. While the dielectric optical fiber waveguide is very efficient in transmitting light, its functionality is somewhat limited by the dielectric materials of the core and cladding and their fixed optical properties (e.g., spectral response) after the fiber drawing fabrication. In addition, most of the available optical fiber components are bulky in size, thus limiting the development of novel compact in-fiber optical devices. Therefore, there is a need to integrate new materials and nanostructures into fiber components for enhanced processing and transmission capabilities, novel functionalities, and compactivity.

Metasurfaces, arrays of subwavelength elements in which each element is configured to control the phase and amplitude of the transmitted, reflected, and scattered light, provide unique ways for advanced light manipulation [9], [10], [11], [12], [13], [14]. Because metasurfaces are by nature flat (typical thickness < 100 nm), conventional three-dimensional optical elements such as lenses or filters could be replaced by flat and low-profile metasurface versions. Integrating these metasurface nanostructures on the fiber facet could facilitate their interactions with the guided core modes of the optical fibers, creating opportunities for the development of novel in-fiber optical applications.

Several initial attempts have been made to fabricate meta-structures on optical fiber for various advanced in-fiber applications including plasmonic sensors [15], [16], [17], [18], [19], metalens [20], [21], [22], diffraction grating [23], amplifier [24], beam diffraction element [25], Bessel beam generation [26], and an efficient fiber coupler [27, 28]. These meta-structures on optical fibers are fabricated by translating advanced on-chip nanofabrication techniques such as electron-beam lithography [19, 29], focused ion beam milling [20], interference lithography [30], self-assembly [31], nano-imprinting/nano transfer technologies [32], [33], [34], and two-photon polymerization direct laser writing technique [21, 22] to the optical fiber platform. In particular, developing an ultracompact wavelength- and polarization-dependent optical fiber metasurface/plasmonic filter and resonant element is particularly important for optical fiber imaging, laser, and sensing applications. A few attempts have been made in this direction, including fabricating a metallic structure to a polymeric membrane on the facet of a hollow-core PCF for a nanoplasmonic filter [35]. However, the successful integration of an ultracompact polarization-dependent metasurface optical filter onto an optical fiber has not been experimentally reported.

In this work, we experimentally demonstrate ultracompact in-fiber polarization-dependent optical filters on the endface of polarization-maintaining photonic crystal fibers (PM-PCFs) and conventional single mode optical fibers by fabricating asymmetric cross-typed nanoslit metasurface array and integrating them onto the optical fibers’ cores. Strongly polarization-dependent transmissions are observed at resonances of metasurface which are designed by the nanostructure’s geometry. The results suggest that asymmetric metasurface-optical fiber could have applications as compact in-fiber wavelength-dependent filters and polarizers for optical fiber imaging and sensing applications.

The metasurface-optical fiber filter consists of periodic negative cross-typed metallic nanostructures with orthogonal slits (Figure 1). A thin layer of gold with thickness of ∼118 nm was deposited on the endface of the optical fiber using magnetron sputtering technique. A customized fiber holder with multiple v-grooves was used to hold and align the optical fibers vertically to ensure that the fiber endfaces were coated with gold uniformly during the sputtering process. These periodic cross-typed metallic slits were then fabricated using the focused ion beam (FIB) milling technique with an accelerated voltage of 30 kV and ion current of 1.5 pA. To avoid the charging effect from the silica glass, silver paste and conducting tape were used to connect between the gold and metallic fiber holder. Two types of optical fibers, conventional single mode fiber and PM-PCFs, were used for fabrication and comparison of the optical response. The PM-PCF used in the experiments consisted of a two-dimensional array of hollow channels running along the entire length of a glass strand with two large circular holes located near the core, thus providing strong birefringence for maintaining the polarization state of the light to interact with the metasurfaces. To fabricate the asymmetric structures on the PM-PCF, special care was taken such that the orthogonal nanoslits were aligned with the slow and fast axes of the fiber during the FIB fabrication. During the fabrication, we aligned the outer circle of the optical fiber using the FIB fabrication program to identify the center of the optical fiber. Based on the center of the optical fiber, we pattern the metasurface structures with precise coordinates with respect to the origin of the fiber. The holey structures in the PM-PCF helped the alignment during the FIB milling. Test patterns were first fabricated in the cladding region to ensure optimal focusing of the ion beam before the actual patterning to the core. Both symmetric and asymmetric metallic nanoslits were fabricated for studying the polarization-dependent transmission properties.

**Figure 1:**
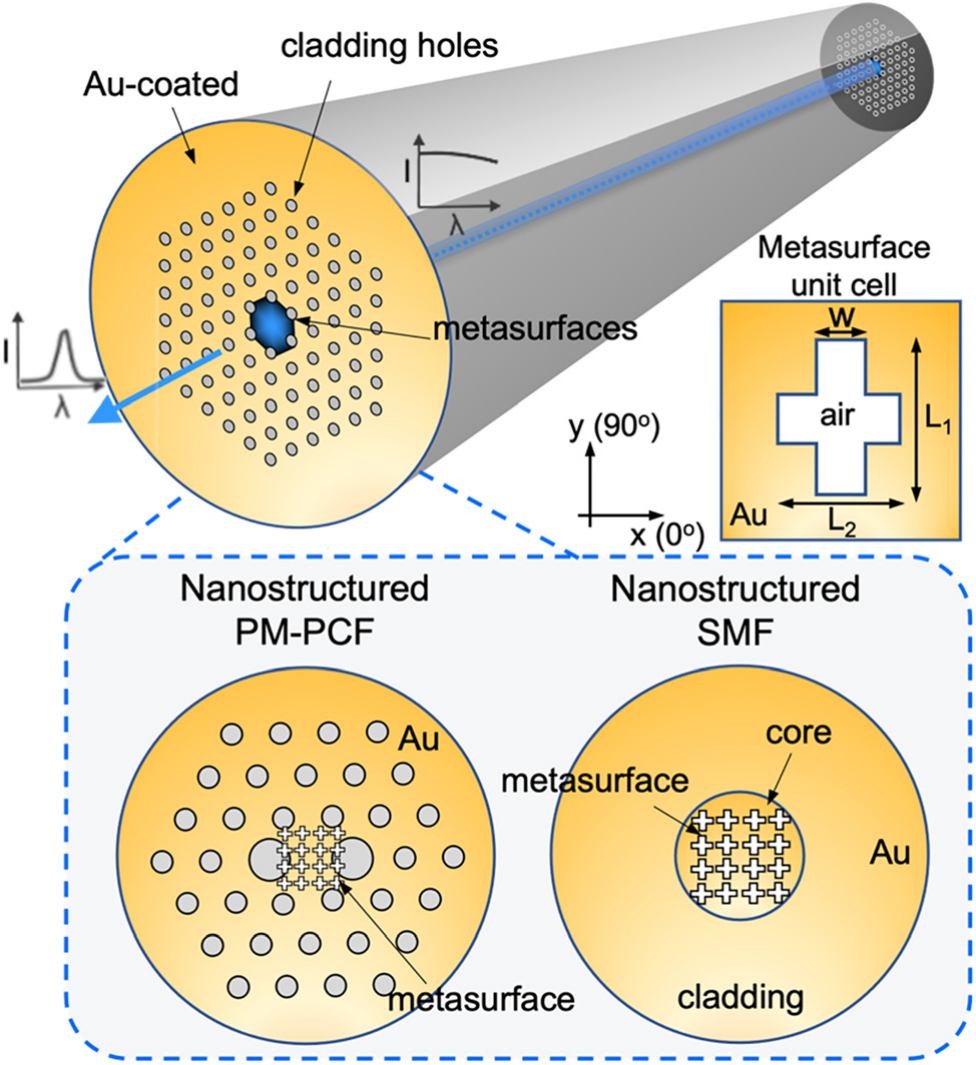
Schematics of the metasurface optical fiber color filters. (Left) Polarization-maintaining photonic crystal fibers (PM-PCFs) nanostructures covering the core region. (Right) Single mode optical fibers (SMF) with nanostructures on the core region, (Inset) Unit element of asymmetric metasurface.

Light coupling to these metallic nanoslits excited plasmonic resonance modes and re-emitted through the transmission, leading to a wavelength-dependent transmission peak. The transmission properties can be designed by adjusting the geometric parameters of nanoslit structures such as slit dimension, array period, and the thickness of gold film. To find the dependence of the transmission peak on the geometric parameters, we numerically studied the optical response of the optical filter using the finite element method (see Method) and performed parametric sweeps on the width and length of the nanocross. The gold layer thickness and periodicity of the array were fixed at 118 and 800 nm. The dependence of transmission with different widths and lengths of the nanocross are shown in Figure 2. For fixed horizontal input polarization state, varying the length of the longer arm of nanocross (*L*_1_) does not change significantly the resonant peak and transmission (width (*w*) and length of shorter arm (*L*_2_) are fixed with 180 and 480 nm) (Figure 2a). In contrast, the transmission peak redshifts linearly as the length of the shorter arm (*L*_2_) (or both *L*_2_ and *L*_1_) increases (Figure 2b and c). Also, the bandwidth and the strength of the resonant peak increase as the length of the shorter arm increases. In addition, transmission is less depended on the width of the nanocross as shown in Figure 2d–f (lengths of longer arm and shorter arm are fixed at 580 and 480 nm, respectively). Since the plasmonic resonance condition is directly related to the width and lengths of the nanocross array, by carefully selecting the geometric factors of the nanocross array, a desired transmission peak position and efficiency can be achieved. Based on these geometric simulations, the final designed structure with a unit nanocross was optimized with a width of 180 nm and the length of the horizontal and vertical arms being 580 and 480 nm long, respectively, such that the transmission peak exhibited high efficiency (∼70%) and was located in the telecommunication band (as indicated with rhombus symbol in Figure 2a, b and f).

**Figure 2:**
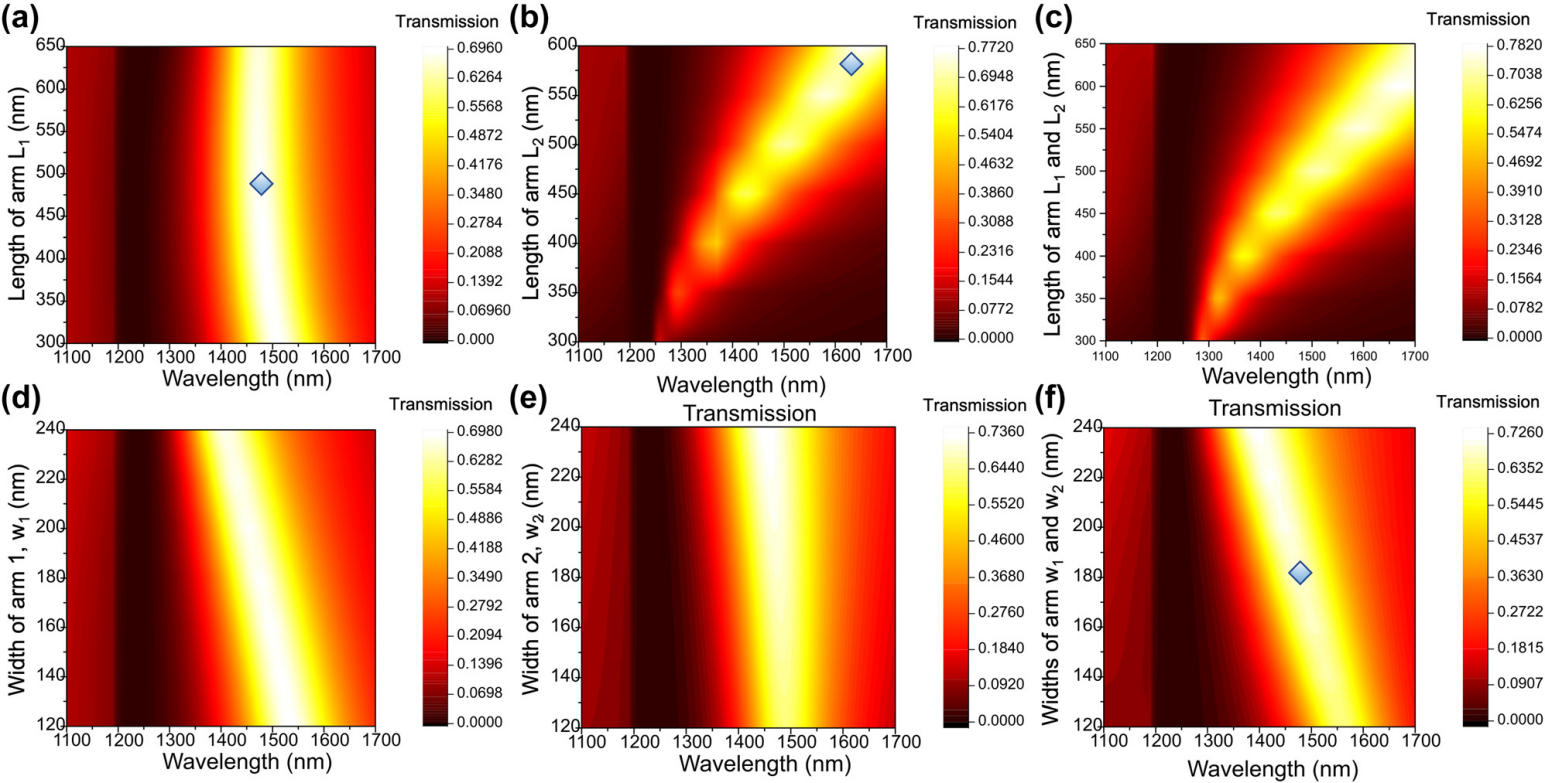
Map of transmission spectra with geometric parameters of nanoslit structures. Wavelength-dependent transmission with varying (a) length of the longer arm of nanocross (*L*_1_) (width and length of shorter arm (*L*_2_) are fixed at 180 and 480 nm), (b) length of the shorter arm of nanocross (*L*_2_) (width and length of longer arm (*L*_2_) are fixed at 180 and 580 nm), (c) and length of both arms of nanocross (*L*_1_ and *L*_2_) (width is fixed at 180 nm). Dependance of transmission peak for varying (d) width of longer arm of nanocross, (e) width of longer arm of nanocross, and (f) width of both arms of nanocross. The rhombus symbols in (a), (b), and (f) indicate the final designed structure of unit nanocross for asymmetric metasurfaces.

The scanning electron microscope (SEM) images of the fabricated structures are depicted in Figure 3. The symmetric nanocross array (unit element width of 176 nm, length of slit of 490 nm) and asymmetric nanocross (unit element width of 182 nm, length of arms of 510 and 419 nm) are fabricated on the core of the PM-PCF with approximately rectangular core dimensions of ∼4.5 × 5.7 μm^2^ (Figure 3a). An asymmetric nanocross (unit element width of 193 nm, length of arm of 577 and 465 nm) are also fabricated on the core of the conventional single mode fiber with a core diameter of 8 μm (Figure 3b).

**Figure 3:**
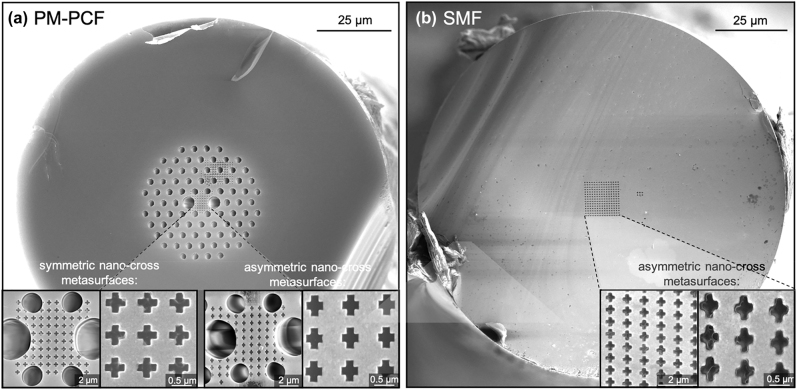
Scanning electron microscopic images of optical fiber metasurface optical filter. SEM images of (a) PM-PCFs with symmetric and asymmetric nanocross metasurfaces fabricated in the rectangular core and (b) conventional single mode fiber with asymmetric nanocross metasurfaces fabricated in the circular core.

A schematic of the setup used for optical measurements is shown in Figure 4a. Light from a supercontinuum laser source (Fianium, 4W) was launched into the fiber sample (total length ∼ 13 cm), taking care to match the numerical aperture and spot size to that of the fundamental core mode. A polarizer and half-wave plate were inserted between the light source and the sample, providing a defined input polarization state. Light transmitted in the output end with metasurface was collected by coupling into multimode fiber to the optical spectrum analyzer. The measured spectrum was compared to that of an unpatterned fiber under the same launching conditions, thus revealing the effect of the metasurface.

**Figure 4:**
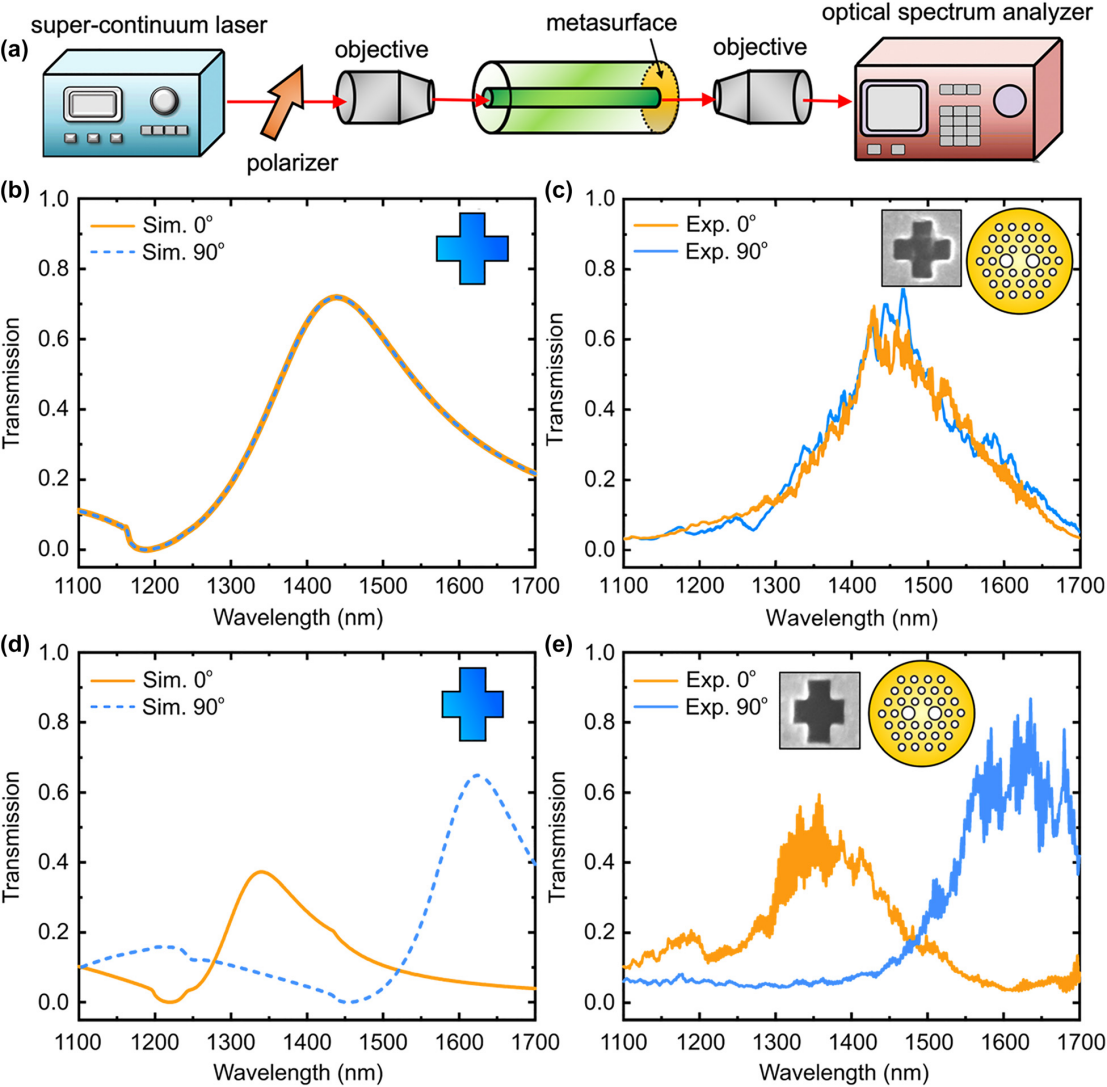
Transmission spectra for PM-PCF metasurface optical filter. (a) Schematic of experimental measurement setup. (b) Simulated and (c) measured transmission spectra for *x*- and *y*-polarization states for PM-PCF with symmetric nanocross metasurfaces. Symmetric nanocross array has unit element wide of 176 nm and length of the slit of 490 nm. (d) Simulated and (e) measured transmission spectra for *x*- and *y*-polarization states for PM-PCF with asymmetric nanocross metasurfaces. Asymmetric nanocross consists of unit element with wide of 182 nm and length of slit of 510 and 419 nm.

To examine the effect of the metasurface, we measured the transmission spectra for *x*- and *y*-polarizations for the PM-PCF with the symmetric nanocross metasurface. The results are shown in Figure 4c. The length of each perpendicular slit is 490 nm, and the width of each slit is 176 nm. Two orthogonally-polarized beams of light along the slow or fast axis of the PM-PCF were launched into the fiber. A clear transmission resonant peak was observed at the wavelength of ∼1460 nm for both horizontal and vertical polarization states with transmission efficiency of ∼70%. Full-wave electromagnetic simulation was performed with the same fabricated metasurface geometry, and the results are shown in Figure 4b. Good agreement was obtained comparing the simulations and the experimental measurements on both the resonant wavelength and the transmission efficiency.

Next, we explored the polarization dependence of the PM-PCF with an asymmetric nanocross metasurface with a unit slit length of 510 nm and width of 419 nm (Figure 3a, right). The metasurface structures are precisely aligned so that the longer arm of the nanocross is along the slow axis of the PM-PCF. Since the polarization state of light can be preserved in the PM-PCF, horizontal or vertical polarization states of the light are sure to interact with the desired axis of the nanocross metasurface. As shown in the measurement results in Figure 4e, distinct transmission resonance peaks located at the wavelengths of 1350 and 1620 nm were observed for the horizontal and vertical polarization states, respectively. Numerical simulations were performed, and the spectral positions of the simulated transmission peaks (1350 and 1630 nm) closely matched those of the experiments (Figure 4). In *x*-polarization, the fundamental core mode of the optical fiber is coupled strongly with the plasmonic resonance with the short arm of the nanocross, leading to a shorter resonant wavelength and lower transmission efficiency compared to the resonant peak in the *y*-polarization state. The slight discrepancy between measurement and simulation might be attributable to the non-uniformity and non-ideal shape of the fabricated nanostructures.

Finally, we investigated the optical filtering properties in a conventional single mode fiber integrated with an asymmetric nanocross metasurface array. In this measurement, unpolarized light was launched into the fiber, and a polarizer was used in the output of the fiber to selectively collect the *x*- or *y*-polarization of the transmitted light. Similar to the case in meta-structured PM-PCF, transmission peaks can be observed at the wavelength of 1400 nm for *x*-polarization state and at 1650 nm for *y*-polarization state (Figure 5). The resonance peaks are located in longer wavelengths than in the case of meta-structured PM-PCF because of the slightly larger fabricated structures (Figure 3b). These results indicate that the metasurface filter can be routinely realized in any conventional optical fiber and can be used as wavelength selective filter and polarizer. It should be noted that the polarization-maintaining PCF used in the experiment can maintain the polarization state even if the light is propagating for long length of fiber or external perturbations exist (e.g. bending of fiber or mechanical vibration); thus meta-structured PM-PCF filter can potentially be used for optical fiber system that requires precise polarization control.

**Figure 5:**
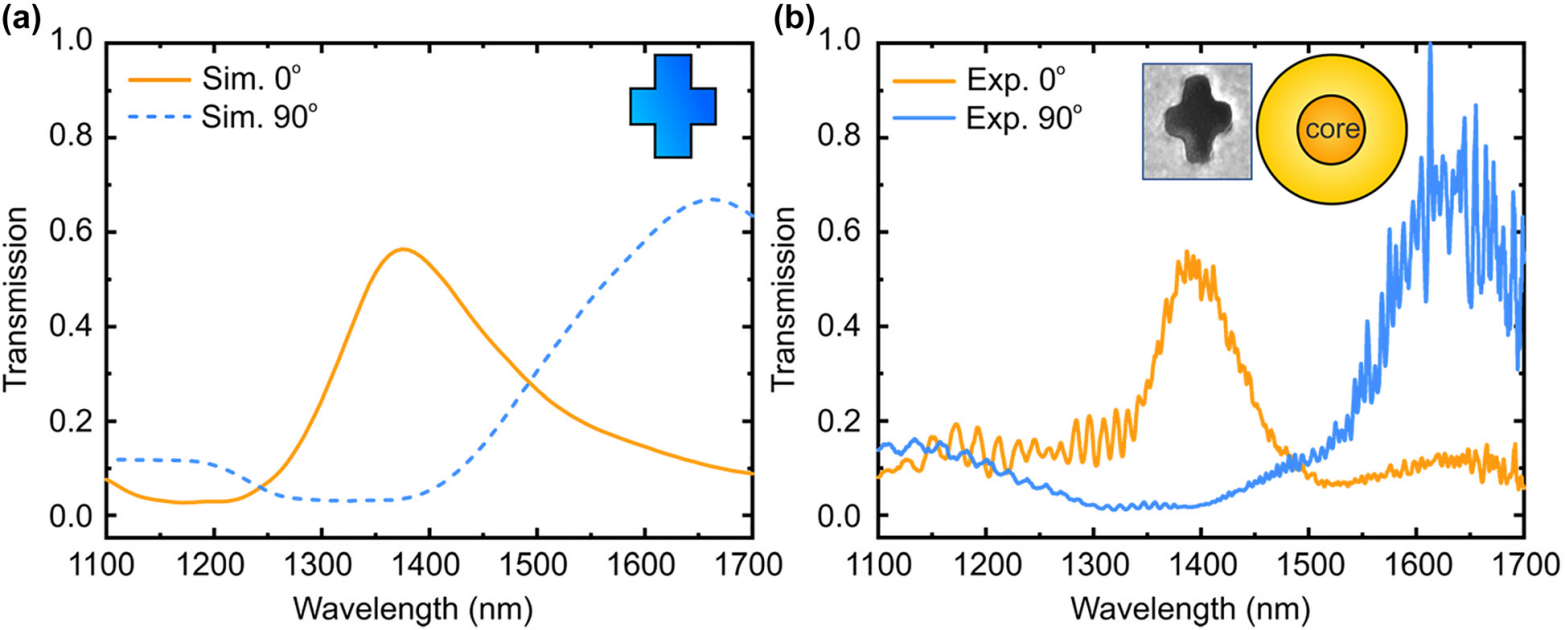
Transmission spectra for SMF metasurface optical filter. (a) Simulated and (b) measured transmission spectra for *x*- and *y*-polarization states for SMF with asymmetric nanocross metasurfaces. Asymmetric nanocross consists of unit element with wide of 193 nm and length of slit of 577 and 465 nm.

## 1 Conclusions

We experimentally demonstrated a polarization-dependent in-fiber optical filter with an ultrathin asymmetric metasurface patterned on the fiber end-facet by the focused ion beam milling technique. Highly polarization- and wavelength-dependent transmission with a transmission efficiency ∼70% in the telecommunication wavelength were observed by launching light into two orthogonal linear polarization states of the fiber. The operation wavelength of the metasurface filter could be widely controlled by nano-engineering the metasurface’s geometry. This work provides a new paradigm for developing nanoscale in-fiber devices such as in-fiber polarization- and wavelength-dependent filters, polarizers, and metalens for emerging optical fiber imaging and sensing applications.

## 2 Methods

### 2.1 Numerical simulation

Simulation of the nanostructures on the fiber was carried out using a full-wave simulation of finite domain time difference (FDTD) software from Lumerical Solutions, Inc. For the simulation, full-wave simulation of unit element was carried out with periodic boundary conditions along the *x*- and *y*-boundaries with mesh size of 1 nm. Full-wave simulation of whole structure on the fiber was carried out with PML boundary condition with mesh size of 2 nm. The polarization-maintaining photonic crystal fiber PM-PCF used was pure silica glass (Thorlabs, PM-1550-01). The PM-PCF consisted of two special holes which were distinguished from all other holes and which reduced the six-fold symmetry to a two-fold one. The presence of two large holes adjacent to the core introduces birefringence in the fiber, leading to a phase index difference between the *x*- and *y*-states. The diameters of the large and small holes are 4.4 and 2.5 µm, respectively.

## Supplementary Material

Supplementary Material
