# Supplementary material for: Polarization-dependent photonic crystal fiber optical filters enabled by asymmetric metasurfaces
Source: Nanophotonics. 2022 Mar 17;11(11):2711–7. doi: 10.1515/nanoph-2022-0001 (PMC11501997; doi:10.1515/nanoph-2022-0001)
Supplement: Supplementary file 1 — Supplementary Material [file j_nanoph-2022-0001_suppl.pdf]

# Polarization-dependent photonic crystal fiber optical filters enabled by asymmetric metasurfaces

Indra Ghimire[1], Jingyi Yang[2], Sudip Gurung[2], Satyendra K. Mishra[1,3], and Ho Wai Howard Lee[1,2*]

[1] Department of Physics and Baylor Research and Innovation Collaborative (BRIC), Baylor University, United States
[2] Department of Physics & Astronomy, University of California, Irvine, CA 92697, United States
[3] Centre for Optics, Photonics, and Lasers (COPL), Université Laval, Canada
*Corresponding author: Ho Wai Howard Lee (Howardhw.lee@uci.edu)

**Abstract:** We demonstrate in-fiber polarization-dependent optical filter by nanopatterning an asymmetric metallic metasurface array on the end-facet of polarization-maintaining photonic-crystal fibers. The asymmetric cross-typed nanoslit metasurface arrays are fabricated on the core of the optical fiber using the focused ion beam milling technique. Highly polarization- and wavelength-dependent transmission with transmission efficiency of ~70 % in the telecommunication wavelength was observed by launching two orthogonal linear-polarization states of light into the fiber. Full-wave electromagnetic simulations are in good agreement with the experimental results. These advanced meta-structured optical fibers can potentially be used as novel ultracompact in-fiber filters, splitters, and polarization converters.

**Deleted:** color

**Keywords:** Metasurface; optical fiber; nanostructure; photonic crystal fiber.

Optical fibers have proven to be an efficient platform for light guiding with low optical loss, leading to wide range of emerging optical applications such as long distance optical communication (*1*), fiber lasers (*2*), in-fiber imaging, sensing, and laser surgery (*3-8*). While the dielectric optical fiber waveguide is very efficient in transmitting light, its functionality is somewhat limited by the dielectric materials of the core and cladding and their fixed optical properties (e.g., spectral response) after the fiber drawing fabrication. In addition, most of the available optical fiber components are bulky in size, thus limiting the development of novel compact in-fiber optical devices. Therefore, there is a need to integrate new materials and nanostructures into fiber components for enhanced processing and transmission capabilities, novel functionalities, and compactivity.

**Formatted:** Highlight

**Deleted:** and

Metasurfaces, arrays of subwavelength elements in which each element is configured to control the phase and amplitude of the transmitted, reflected, and scattered light, provide unique ways for advanced light manipulation (*9-14*). Because metasurfaces are by nature flat (typical thickness <100 nm), conventional three-dimensional optical elements such as lenses or filters could be replaced by flat and low-profile metasurface versions. Integrating these metasurface nanostructures on the fiber facet could facilitate their interactions with the guided core modes of the optical fibers, creating opportunities for the development of novel in-fiber optical applications.

Several initial attempts have been made to fabricate meta-structures on optical fiber for various advanced in-fiber applications including plasmonic sensors (*15-19*), metalens (*20-22*), diffraction grating (*23*), amplifier (*24*), beam diffraction element (*25*), Bessel beam generation (*26*), and an efficient fiber coupler (*27, 28*). These meta-structures on optical fibers are fabricated by translating advanced on-chip nanofabrication techniques such as electron-beam lithography (*19, 29*), focused ion beam milling (*20*), interference lithography (*30*), self-assembly (*31*), nano-imprinting/nano transfer technologies (*32-34*), and two-photon polymerization direct laser writing technique (*21, 22*) to the optical fiber platform. In particular, developing an ultracompact wavelength- and polarization-dependent optical fiber metasurface/plasmonic filter and resonant element is particularly important for optical fiber imaging, laser, and sensing applications. A few attempts have been made in this direction, including fabricating a metallic structure to a polymeric membrane on the facet of a hollow-core PCF for a nano-plasmonic filter (*35*). However, the successful integration of an ultracompact polarization-dependent metasurface optical filter onto an optical fiber has not been experimentally reported.

In this work, we experimentally demonstrate ultracompact in-fiber polarization-dependent optical filters on the endface of polarization-maintaining photonic crystal fibers (PM-PCFs) and conventional single mode optical fibers by fabricating asymmetric cross-typed nanoslit metasurface array and integrating them onto the optical fibers' cores. Strongly polarization-dependent transmissions are observed at resonances of metasurface which are designed by the nanostructure's geometry. The results suggest that asymmetric metasurface-optical fiber could have applications as compact in-fiber wavelength-dependent filters and polarizers for optical fiber imaging and sensing applications.

Deleted: color

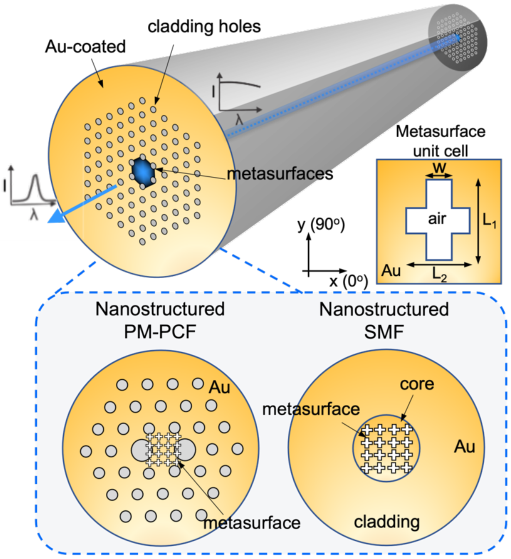


**Figure 1**. **Schematics of the metasurface optical fiber color filters.** (Left) Polarization-maintaining photonic crystal fibers (PM-PCFs) nanostructures covering the core region. (Right) Single mode optical fibers (SMF) with nanostructures on the core region, (Inset) Unit element of asymmetric metasurface.

The metasurface-optical fiber filter consists of periodic negative cross-typed metallic nanostructures with orthogonal slits (Fig. 1). A thin layer of gold with thickness of ~ 118 nm was deposited on the endface of the optical fiber using magnetron sputtering technique. A customized fiber holder with multiple v-grooves was used to hold and align the optical fibers vertically to ensure that the fiber endfaces were coated with gold uniformly during the sputtering process. These periodic cross-typed metallic slits were then fabricated using the focused ion beam (FIB) milling technique with an accelerated voltage of 30 kV and ion current of 1.5 pA. To avoid the charging effect from the silica glass, silver paste and conducting tape were used to connect between the gold and metallic fiber holder. Two types of optical fibers, conventional single mode fiber and PM-PCFs, were used for fabrication and comparison of the optical response. The PM-PCF used in the experiments consisted of a two-dimensional array of hollow channels running along the entire length of a glass strand with two large circular holes located near the core, thus providing strong birefringence for maintaining the polarization state of the light to interact with the metasurfaces. To fabricate the asymmetric structures on the PM-PCF, special care was taken such that the orthogonal nano-slits were aligned with the slow and fast axes of the fiber during the FIB fabrication. During the fabrication, we aligned the outer circle of the optical fiber using the FIB fabrication program to identify the center of the optical fiber. Based on the center of the optical fiber, we pattern the metasurface structures with precise coordinates with respect to the origin of the fiber. The holey structures in the PM-PCF helped the alignment during the FIB milling. Test patterns

Deleted: color

Formatted: Highlight

Formatted: Highlight

were first fabricated in the cladding region to ensure optimal focusing of the ion beam before the actual patterning to the core. Both symmetric and asymmetric metallic nano-slits were fabricated for studying the polarization-dependent transmission properties.

Light coupling to these metallic nano-slits excited plasmonic resonance modes and re-emitted through the transmission, leading to a wavelength-dependent transmission peak. The transmission properties can be designed by adjusting the geometric parameters of nano-slit structures such as slit dimension, array period, and the thickness of gold film. To find the dependence of the transmission peak on the geometric parameters, we numerically studied the optical response of the optical filter using the finite element method (see Method) and performed parametric sweeps on the width and length of the nano-cross. The gold layer thickness and periodicity of the array were fixed at 118 nm and 800 nm . The dependence of transmission with different widths and lengths of the nano-cross are shown in Fig. 2. For fixed horizontal input polarization state, varying the length of the longer arm of nano-cross ($L_1$) does not change significantly the resonant peak and transmission (width (w) and length of shorter arm ($L_2$) are fixed with 180 nm and 480 nm) (Fig. 2a). In contrast, the transmission peak redshifts linearly as the length of the shorter arm ($L_2$) (or both $L_2$ and $L_1$) increases (Fig. 2b,c). Also, the bandwidth and the strength of the resonant peak increase as the length of the shorter arm increases. In addition, transmission is less depended on the width of the nano-cross as shown in Fig. 2d-f (lengths of longer arm and shorter arm are fixed at 580 nm and 480 nm, respectively). Since the plasmonic resonance condition is directly related to the width and lengths of the nano-cross array, by carefully selecting the geometric factors of the nano-cross array, a desired transmission peak position and efficiency can be achieved. Based on these geometric simulations, the final designed structure with a unit nano-cross was optimized with a width of 180 nm and the length of the horizontal and vertical arms being 580 nm and 480 nm long respectively such that the transmission peak exhibited high efficiency (~70%) and was located in the telecommunication band (as indicated with rhombus symbol in Fig. 2 a,b,f).

Deleted: color

Formatted: Highlight

Formatted: Highlight

Formatted: Highlight

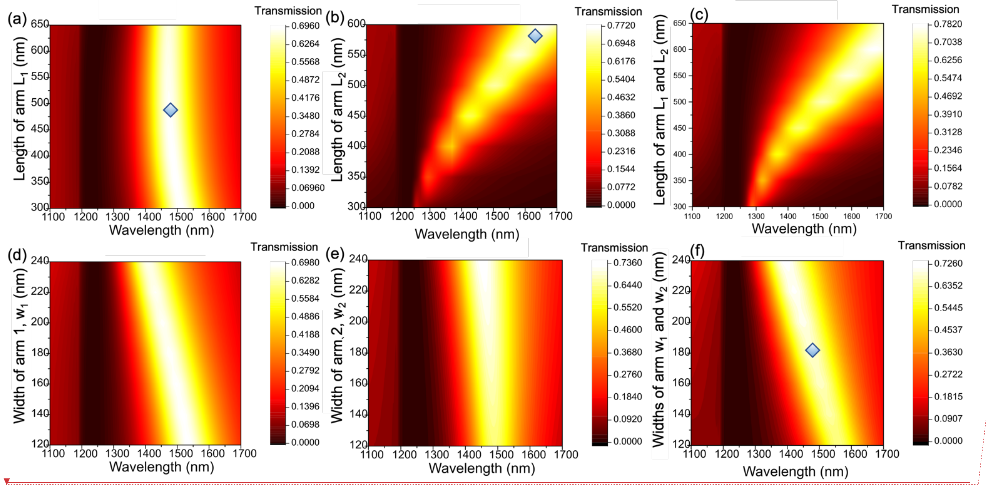


Deleted:

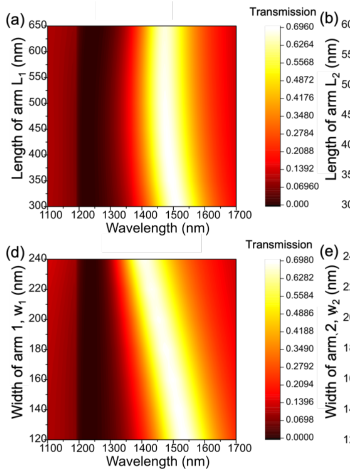

**Figure 2: Map of transmission spectra with geometric parameters of nano-slit structures.** Wavelength-dependent transmission with varying (a) length of the longer arm of nano-cross ($L_1$) (width and length of shorter arm ($L_2$) are fixed at 180 nm and 480 nm), (b) length of the shorter arm of nano-cross ($L_2$) (width and length of longer arm ($L_2$) are fixed at 180 nm and 580 nm), (c) and length of both arms of nano-cross ($L_1$ and $L_2$) (width is fixed at 180 nm). Dependance of transmission peak for varying (d) width of longer arm of nano-cross, (e) width of longer arm of nano-cross, and (f) width of both arms of nano-cross. The rhombus symbols in Fig. 2 a,b,f indicate the final designed structure of unit nano-cross for asymmetric metasurfaces.

Formatted: Highlight

The scanning electron microscope (SEM) images of the fabricated structures are depicted in Fig. 3. The symmetric nano-cross array (unit element width of 176 nm, length of slit of 490 nm) and asymmetric nano-cross (unit element width of 182 nm, length of arms of 510 nm and 419 nm) are fabricated on the core of the PM-PCF with approximately rectangular core dimensions of ~ 4.5 x 5.7 μm$^2$ (Fig. 3a). An asymmetric nano-cross (unit element width of 193 nm, length of arm of 577 nm and 465 nm) are also fabricated on the core of the conventional single mode fiber with a core diameter of 8 μm (Fig. 3b).

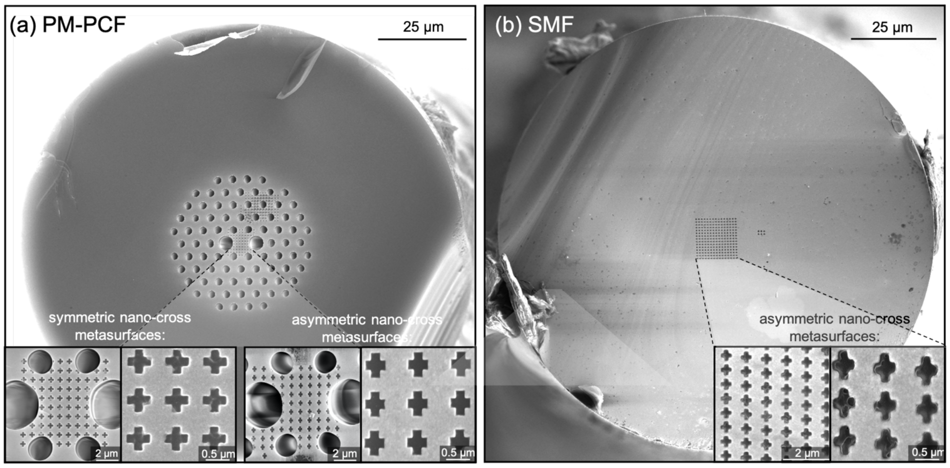


**Figure 3: Scanning electron microscopic images of optical fiber metasurface optical filter.** SEM images of (a) PM-PCFs with symmetric and asymmetric nano-cross metasurfaces fabricated in the rectangular core and (b) conventional single mode fiber with asymmetric nano-cross metasurfaces fabricated in the circular core.

Deleted: color

A schematic of the setup used for optical measurements is shown in Fig. 4a. Light from a supercontinuum laser source (Fianium, 4W) was launched into the fiber sample (total length ~ 13 cm), taking care to match the numerical aperture and spot size to that of the fundamental core mode. A polarizer and half-wave plate were inserted between the light source and the sample, providing a defined input polarization state. Light transmitted in the output end with metasurface was collected by coupling into multimode fiber to the optical spectrum analyzer. The measured spectrum was compared to that of an unpatterned fiber under the same launching conditions, thus revealing the effect of the metasurface.

To examine the effect of the metasurface, we measured the transmission spectra for x- and y-polarizations for the PM-PCF with the symmetric nano-cross metasurface. The results are shown in Fig. 4c. The length of each perpendicular slit is 490 nm, and the width of each slit is 176 nm. Two orthogonally-polarized beams of light along the slow or fast axis of the PM-PCF were launched into the fiber. A clear transmission resonant peak was observed at the wavelength of ~1460 nm for both horizontal and vertical polarization states with transmission efficiency of ~70%. Full-wave electromagnetic simulation was performed with the same fabricated metasurface geometry, and the results are shown in Fig. 4b. Good agreement was obtained comparing the simulations and the experimental measurements on both the resonant wavelength and the transmission efficiency.

Next, we explored the polarization dependence of the PM-PCF with an asymmetric nano-cross metasurface with a unit slit length of 510 nm and width of 419 nm (Fig. 3a, right). The metasurface structures are precisely aligned so that the longer arm of the nano-cross is along the slow axis of the PM-PCF. Since the polarization state of light can be preserved in the PM-PCF, horizontal or vertical polarization states of the light are sure to interact with the desired axis of the nano-cross metasurface. As shown in the measurement results in Fig. 4e, distinct transmission resonance peaks located at the wavelengths of 1350 nm and 1620 nm were observed for the horizontal and vertical polarization states, respectively. Numerical simulations were performed, and the spectral positions of the simulated transmission

peaks (1350 nm and 1630 nm) closely matched those of the experiments (Fig. 4). In x-polarization, the fundamental core mode of the optical fiber is coupled strongly with the plasmonic resonance with the short arm of the nano-cross, leading to a shorter resonant wavelength and lower transmission efficiency compared to the resonant peak in the y-polarization state. The slight discrepancy between measurement and simulation might be attributable to the non-uniformity and non-ideal shape of the fabricated nanostructures.

Finally, we investigated the optical filtering properties in a conventional single mode fiber integrated with an asymmetric nano-cross metasurface array. In this measurement, unpolarized light was launched into the fiber, and a polarizer was used in the output of the fiber to selectively collect the x- or y-polarization of the transmitted light. Similar to the case in meta-structured PM-PCF, transmission peaks can be observed at the wavelength of 1400 nm for x-polarization state and at 1650 nm for y-polarization state (Fig. 5). The resonance peaks are located in longer wavelengths than in the case of meta-structured PM-PCF because of the slightly larger fabricated structures (Fig. 3b). These results indicate that the metasurface filter can be routinely realized in any conventional optical fiber and can be used as wavelength selective filter and polarizer. It should be noted that the polarization-maintaining PCF used in the experiment can maintain the polarization state even if the light is propagating for long length of fiber or external perturbations exist (e.g. bending of fiber or mechanical vibration); thus meta-structured PM-PCF filter can potentially be used for optical fiber system that requires precise polarization control.

Deleted: color

Deleted: color

Formatted: Highlight

Formatted: Highlight

Formatted: Highlight

Formatted: Highlight

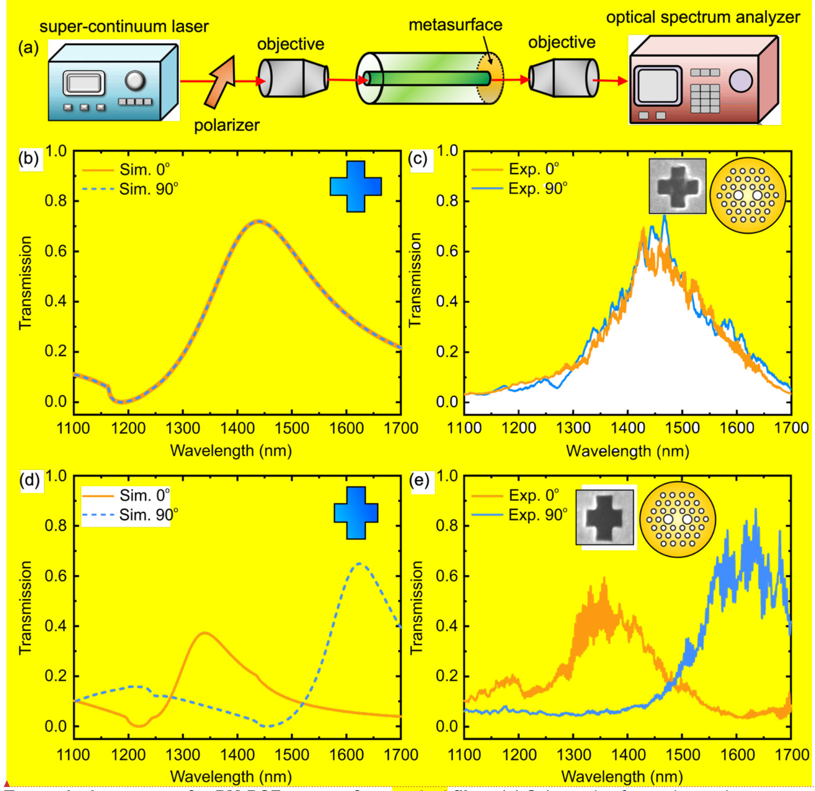


**Figure 4. Transmission spectra for PM-PCF metasurface optical filter.** (a) Schematic of experimental measurement setup. (b) Simulated and (c) measured transmission spectra for x- and y-polarization states for PM-PCF with symmetric nano-cross metasurfaces. Symmetric nano-cross array has unit element wide of 176 nm and length of the slit of 490 nm. (d) Simulated and

Deleted: color

(e) measured transmission spectra for x- and y-polarization states for PM-PCF with asymmetric nano-cross metasurfaces. Asymmetric nano-cross consists of unit element with wide of 182 nm and length of slit of 510 nm and 419 nm.

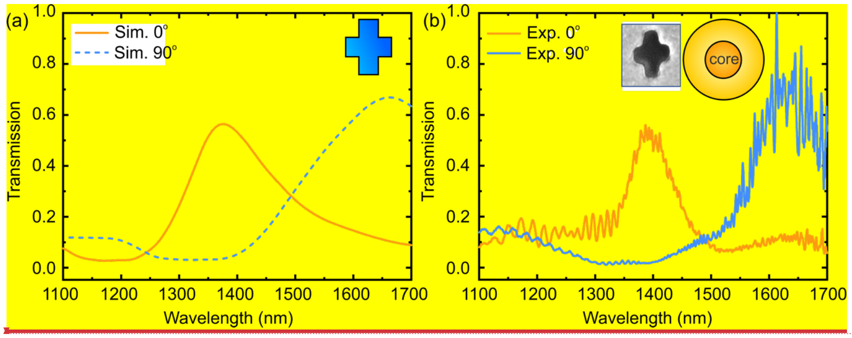


**Figure 5. Transmission spectra for SMF metasurface optical filter.** (a) Simulated and (b) measured transmission spectra for x- and y-polarization states for SMF with asymmetric nano-cross metasurfaces. Asymmetric nano-cross consists of unit element with wide of 193 nm and length of slit of 577 nm and 465 nm.

**Deleted:**

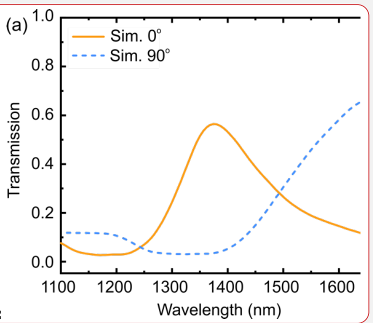


**Formatted:** Highlight

**Deleted:** color

## Conclusions

We experimentally demonstrated a polarization-dependent in-fiber optical filter with an ultrathin asymmetric metasurface patterned on the fiber end-facet by the focused ion beam milling technique. Highly polarization- and wavelength-dependent transmission with a transmission efficiency ~ 70 % in the telecommunication wavelength were observed by launching light into two orthogonal linear polarization states of the fiber. The operation wavelength of the metasurface filter could be widely controlled by nano-engineering the metasurface's geometry. This work provides a new paradigm for developing nanoscale in-fiber devices such as in-fiber polarization- and wavelength-dependent filters, polarizers, and metalens for emerging optical fiber imaging and sensing applications.

**Deleted:** color

**Deleted:** color

## Methods

*Numerical simulation*. Simulation of the nanostructures on the fiber was carried out using a full-wave simulation of Finite Domain Time Difference (FDTD) software from Lumerical Solutions, Inc. For the simulation, full-wave simulation of unit element was carried out with periodic boundary conditions along the x- and y- boundaries with mesh size of 1 nm. Full-wave simulation of whole structure on the fiber was carried out with PML boundary condition with mesh size of 2 nm. The polarization-maintaining photonic crystal fiber PM-PCF used was pure silica glass (Thorlabs, PM-1550-01). The PM-PCF consisted of two special holes which were distinguished from all other holes and which reduced the six-fold symmetry to a two-fold one. The presence of two large holes adjacent to the core introduces birefringence in the fiber, leading to a phase index difference between the *x*- and *y*- states. The diameters of the large and small holes are 4.4 µm and 2.5 µm, respectively.

## Acknowledgments

This work was supported by AFOSR (FA9550-21-1-02204). The authors acknowledge the Center for Microscopy and Imaging (CMI) at Baylor University for technical support during the microscopy and image analysis.

## References


1. P. J. Winzer, D. T. Neilson, A. R. Chraplyvy, Fiber-optic transmission and networking: the previous 20 and the next 20 years [Invited]. *Opt. Express* **26**, 24190-24239 (2018).
2. P. D. Dragic, M. Cavillon, J. Ballato, Materials for optical fiber lasers: A review. *Appl Phys Rev* **5**, 041301 (2018).
3. A. Urrutia, I. D. Villar, P. Zubiate, C. R. Zamarreño, A Comprehensive Review of Optical Fiber Refractometers: Toward a Standard Comparative Criterion. *Laser & Photonics Reviews* **13**, 1900094 (2019).
4. H. K. Lee, J. Choo, J. Kim, Multiplexed Passive Optical Fiber Sensor Networks for Water Level Monitoring: A Review. *Sensors (Basel)* **20**, (2020).
5. A. Orth, M. Ploschner, E. R. Wilson, I. S. Maksymov, B. C. Gibson, Optical fiber bundles: Ultra-slim light field imaging probes. *Sci Adv* **5**, eaav1555 (2019).
6. A. B. Socorro-Leránoz, D. Santano, I. D. Villar, I. R. Matias, Trends in the design of wavelength-based optical fibre biosensors. *Biosensors and Bioelectronics: X* **1**, 100015 (2019).
7. T. Čižmár, K. Dholakia, Shaping the light transmission through a multimode optical fibre: complex transformation analysis and applications in biophotonics. *Opt. Express* **19**, 18871-18884 (2011).
8. S. F. Ioannis N. Papadopoulos, Christophe Moser, and Demetri Psaltis, High-resolution, lensless endoscope based on digital scanning through a multimode optical fiber. *Optical Society of America*, (2013).
9. A. Li, S. Singh, D. Sievenpiper, Metasurfaces and their applications. *Nanophotonics* **7**, 989-1011 (2018).
10. A. V. Kildishev, A. Boltasseva, V. M. Shalaev, Planar photonics with metasurfaces. *Science* **339**, 1232009 (2013).
11. N. Meinzer, W. L. Barnes, I. R. Hooper, Plasmonic meta-atoms and metasurfaces. *Nature Photonics* **8**, 889-898 (2014).
12. H. T. Chen, A. J. Taylor, N. Yu, A review of metasurfaces: physics and applications. *Rep Prog Phys* **79**, 076401 (2016).
13. D. Lin, P. Fan, E. Hasman, M. L. Brongersma, Dielectric gradient metasurface optical elements. *Science* **345**, 298-302 (2014).
14. S. Sun, K. Y. Yang, C. M. Wang, T. K. Juan, W. T. Chen, C. Y. Liao, Q. He, S. Xiao, W. T. Kung, G. Y. Guo, L. Zhou, D. P. Tsai, High-efficiency broadband anomalous reflection by gradient meta-surfaces. *Nano Lett.* **12**, 6223-6229 (2012).
15. Y. Liang, Z. Yu, L. Li, T. Xu, A self-assembled plasmonic optical fiber nanoprobe for label-free biosensing. *Sci Rep* **9**, 7379 (2019).
16. P. Jia, J. Yang, Integration of large-area metallic nanohole arrays with multimode optical fibers for surface plasmon resonance sensing. *Applied Physics Letters* **102**, (2013).
17. H. Nguyen, F. Sidiroglou, S. F. Collins, T. J. Davis, A. Roberts, G. W. Baxter, A localized surface plasmon resonance-based optical fiber sensor with sub-wavelength apertures. *Applied Physics Letters* **103**, (2013).
18. S. Feng, S. Darmawi, T. Henning, P. J. Klar, X. Zhang, A miniaturized sensor consisting of concentric metallic nanorings on the end facet of an optical fiber. *Small* **8**, 1937-1944 (2012).
19. N. Wang, M. Zeisberger, U. Hübner, M. A. Schmidt, Nanotrimer enhanced optical fiber tips implemented by electron beam lithography. *Optical Materials Express* **8**, 2246-2255 (2018).
20. J. Yang, I. Ghimire, P. C. Wu, S. Gurung, C. Arndt, D. P. Tsai, H. W. H. Lee, Photonic crystal fiber metalens. *Nanophotonics* **8**, 443-449 (2019).
21. W. Hadibrata, H. Wei, S. Krishnaswamy, K. Aydin, Inverse Design and 3D Printing of a Metalens on an Optical Fiber Tip for Direct Laser Lithography. *Nano Lett.* **21**, 2422-2428 (2021).
22. M. Plidschun, H. Ren, J. Kim, R. Forster, S. A. Maier, M. A. Schmidt, Ultrahigh numerical aperture meta-fibre for flexible optical trapping. *Light Sci Appl* **10**, 57 (2021).
23. V. Savinov, N. I. Zheludev, High-quality metamaterial dispersive grating on the facet of an optical fiber. *Applied Physics Letters* **111**, (2017).
24. H. E. Arabi, H. E. Joe, T. Nazari, B. K. Min, K. Oh, A high throughput supra-wavelength plasmonic bull's eye photon sorter spatially and spectrally multiplexed on silica optical fiber facet. *Opt Express* **21**, 28083-28094 (2013).
25. M. Principe, M. Consales, A. Micco, A. Crescitelli, G. Castaldi, E. Esposito, V. La Ferrara, A. Cutolo, V. Galdi, A. Cusano, Optical fiber meta-tips. *Light Sci Appl* **6**, e16226 (2017).

26. S. Kang, H.-E. Joe, J. Kim, Y. Jeong, B.-K. Min, K. Oh, Subwavelength plasmonic lens patterned on a composite optical fiber facet for quasi-one-dimensional Bessel beam generation. *Applied Physics Letters* **98**, (2011).
27. O. Yermakov, H. Schneidewind, U. Hübner, T. Wieduwilt, M. Zeisberger, A. Bogdanov, Y. Kivshar, M. A. Schmidt, Nanostructure-Empowered Efficient Coupling of Light into Optical Fibers at Extraordinarily Large Angles. *Acs Photonics* **7**, 2834-2841 (2020).
28. N. Wang, M. Zeisberger, U. Hübner, M. A. Schmidt, Boosting Light Collection Efficiency of Optical Fibers Using Metallic Nanostructures. *Acs Photonics* **6**, 691-698 (2019).
29. M. Zeisberger, H. Schneidewind, U. Hübner, T. Wieduwilt, M. Plidschun, M. A. Schmidt, Plasmonic Metalens-Enhanced Single-Mode Fibers: A Pathway Toward Remote Light Focusing. *Advanced Photonics Research*, 2100100 (2021).
30. X. Yang, N. Ileri, C. C. Larson, T. C. Carlson, J. A. Britten, A. S. Chang, C. Gu, T. C. Bond, Nanopillar array on a fiber facet for highly sensitive surface-enhanced Raman scattering. *Opt. Express* **20**, 24819-24826 (2012).
31. H. H. Jeong, N. Erdene, J. H. Park, D. H. Jeong, H. Y. Lee, S. K. Lee, Real-time label-free immunoassay of interferon-gamma and prostate-specific antigen using a Fiber-Optic Localized Surface Plasmon Resonance sensor. *Biosens. Bioelectron.* **39**, 346-351 (2013).
32. G. Kostovski, U. Chinnasamy, S. Jayawardhana, P. R. Stoddart, A. Mitchell, Sub-15nm optical fiber nanoimprint lithography: A parallel, self-aligned and portable approach. *Adv. Mater.* **23**, 531-535 (2011).
33. D. J. Lipomi, R. V. Martinez, M. A. Kats, S. H. Kang, P. Kim, J. Aizenberg, F. Capasso, G. M. Whitesides, Patterning the tips of optical fibers with metallic nanostructures using nanoskiving. *Nano Lett.* **11**, 632-636 (2011).
34. G. Shambata), J. Provine, K. Rivoire, T. Sarmiento, J. Harris, J. Vučković, Optical fiber tips functionalized with semiconductor photonic crystal cavities. *Appl. Phys. Lett.* **99**, 191102 (2011).
35. P. Reader-Harris, A. Di Falco, Nanoplasmonic Filters for Hollow Core Photonic Crystal Fibers. *Acs Photonics* **1**, 985-989 (2014).
